# Association of Variation in US County-Level Rates of Liver Surgical Resection for Colorectal Liver Metastasis With Poverty Rates in 2010

**DOI:** 10.1001/jamanetworkopen.2023.0797

**Published:** 2023-02-27

**Authors:** George Molina, Mengyuan Ruan, Stuart R. Lipsitz, Hari S. Iyer, Michael J. Hassett, Mary E. Brindle, Quoc-Dien Trinh

**Affiliations:** 1 Division of Surgical Oncology, Department of Surgery, Brigham and Women’s Hospital, Boston, Massachusetts; 2 Center for Surgery and Public Health, Department of Surgery, Brigham and Women’s Hospital, Boston, Massachusetts; 3 Dana-Farber/Brigham and Women’s Cancer Center, Boston, Massachusetts; 4 Ariadne Labs, Brigham and Women’s Hospital, Harvard T.H. Chan School of Public Health, Boston, Massachusetts; 5 Section of Epidemiology and Health Outcomes, Rutgers-Cancer Institute of New Jersey, New Brunswick; 6 Department of Medical Oncology, Dana-Farber Cancer Institute, Boston, Massachusetts; 7 Division of Urological Surgery, Department of Surgery, Brigham and Women’s Hospital, Boston, Massachusetts

## Abstract

**Importance:**

Among patients with colorectal liver metastasis (CRLM) who are eligible for curative-intent liver surgical resection, only half undergo liver metastasectomy. It is currently unclear how rates of liver metastasectomy vary geographically in the US. Geographic differences in county-level socioeconomic characteristics may, in part, explain variability in the receipt of liver metastasectomy for CRLM.

**Objective:**

To describe county-level variation in the receipt of liver metastasectomy for CRLM in the US and its association with poverty rates.

**Design, Setting, and Participants:**

This ecological, cross-sectional, and county-level analysis was conducted using data from the Surveillance, Epidemiology, and End Results Research Plus database. The study included the county-level proportion of patients who had colorectal adenocarcinoma diagnosed between January 1, 2010, and December 31, 2018, underwent primary surgical resection, and had liver metastasis without extrahepatic metastasis. The county-level proportion of patients with stage I colorectal cancer (CRC) was used as a comparator. Data analysis was performed on March 2, 2022.

**Exposures:**

County-level poverty in 2010 obtained from the US Census (proportion of county population below the federal poverty level).

**Main Outcomes and Measures:**

The primary outcome was county-level odds of liver metastasectomy for CRLM. The comparator outcome was county-level odds of surgical resection for stage I CRC. Multivariable binomial logistic regression accounting for clustering of outcomes within a county via an overdispersion parameter was used to estimate the county-level odds of receiving a liver metastasectomy for CRLM associated with a 10% increase in poverty rate.

**Results:**

In the 194 US counties included in this study, there were 11 348 patients. At the county level, the majority of the population was male (mean [SD], 56.9% [10.2%]), White (71.9% [20.0%]), and aged between 50 and 64 (38.1% [11.0%]) or 65 and 79 (33.6% [11.4%]) years. The adjusted odds of undergoing a liver metastasectomy was lower in counties with higher poverty in 2010 (per 10% increase; odds ratio, 0.82 [95% CI, 0.69-0.96]; *P* = .02). County-level poverty was not associated with receipt of surgery for stage I CRC. Despite the difference in rates of surgery (mean county-level rates were 0.24 for liver metastasectomy for CRLM and 0.75 for surgery for stage I CRC), the variance at the county-level for these 2 surgical procedures was similar (*F*_370, 193_ = 0.81; *P* = .08).

**Conclusions and Relevance:**

The findings of this study suggest that higher poverty was associated with lower receipt of liver metastasectomy among US patients with CRLM. Surgery for a more common and less complex cancer comparator (ie, stage I CRC) was not observed to be associated with county-level poverty rates. However, county-level variation in surgical rates was similar for CRLM and stage I CRC. These findings further suggest that access to surgical care for complex gastrointestinal cancers such as CRLM may be partially influenced by where patients live.

## Key Points

**Question:**

Do county-level rates of liver metastasectomy for colorectal liver metastasis (CRLM) vary geographically in the US based on county-level socioeconomic characteristics?

**Findings:**

In this cross-sectional study of 194 US counties and 11 348 patients, the adjusted odds of undergoing liver metastasectomy for CRLM was lower in counties with higher poverty in 2010. However, county-level poverty was not associated with receipt of surgery for stage I colorectal cancer.

**Meaning:**

The results of this study suggest that access to surgical care for complex gastrointestinal cancers such as CRLM in the US may be partially affected by where patients live.

## Teaser

This cross-sectional study uses Surveillance, Epidemiology, and End Results Research Plus data to examine the association of US county-level variation in receipt of liver metastasectomy for colorectal liver metastasis with poverty rates in 2010.

## Introduction

Advances in the surgical treatment of colorectal liver metastasis (CRLM) are potentially being underutilized and benefits to patients may not be distributed equitably.^1,2,3^ Colorectal liver metastasis is unlike other stage IV cancers in that curative-intent surgical resection can result in substantial survival benefit. In highly selected patients, more than 50% are alive at 5 years; in patients with wild-type RAS, 5-year overall survival has been reported to be as high as 63.3%.^4,5,6,7,8^ Despite its progress in prolonging survival among patients with CRLM, this treatment is potentially being underutilized based on available data and estimates.^1,2,3,9^ It is estimated that about half all of patients with CRLM who are potentially eligible for a liver metastasectomy actually receive treatment despite curative-intent liver surgical resection being included in the National Comprehensive Cancer Network guidelines.^1,10,11,12^

Multiple barriers may contribute to disparities in undergoing a liver metastasectomy for CRLM. Previous studies have reported that the setting of care matters. Patients were more likely to undergo a liver metastasectomy at hospitals treating wealthier patients and with a higher volume of related cancer care.^2,10,13^ In California, multiple sociodemographic and health care access factors were associated with undergoing a surgical treatment of CRLM, including race and ethnicity, insurance status, facility, and where patients resided.^3,14^ Although Black patients with CRLM were found to have lower overall survival, this difference between White and Black patients disappeared when accounting for the receipt of liver metastasectomy.^3^ Residential segregation has been reported as a potential factor associated with inequitable access to health care and to cancer treatment for multiple cancers, including colorectal, breast, pancreatic, and prostate.^15,16,17,18,19^ The association between sociodemographic factors and receipt of cancer care has been shown previously for cancers other than CRLM.^20,21,22,23^ Therefore, determining if geographic differences in county-level socioeconomic characteristics may, in part, explain variability in the receipt of liver metastasectomy for CRLM is important to begin to develop interventions to address issues of access.

The objective of this study was to describe the regional variation in undergoing a liver metastasectomy for CRLM by estimating the association between rates of liver surgical resection for CRLM and socioeconomic characteristics at the county level. We hypothesized that counties with higher poverty would have lower odds of the county-level proportion of patients undergoing liver metastasectomy for CRLM.

## Methods

The Brigham and Women’s Institutional Review Board approved this cross-sectional study. The requirement for informed consent was waived owing to the use of deidentified data. The Strengthening the Reporting of Observational Studies in Epidemiology (STROBE) reporting guideline was followed.

### Data Source

This study used data from the Surveillance, Epidemiology, and End Results (SEER) Research Plus database (18 registries; for November 2020 sub 2000-2018) that were linked to county attributes. This database provides data at the county level and allows the identification of patients according to county of residence. However, SEER Research Plus limits the ability to report at the patient level and only allows for analysis and reporting at the county level. County-level sociodemographic data were obtained from the 2018 County Health Rankings Key Findings generated by the University of Wisconsin Population Health Institute.^24^ These county-level social demographic data included the following for the years listed: percentage of the population below the poverty rate (2010), residential segregation (Black/White index, 2012-2016), percentage of uninsured adults (2015), income inequality (2012-2016), and percentage of the county population aged 65 years or older (2016; eTable 1 in Supplement 1). Mean population density for counties was obtained from the US Census Bureau 2014 to 2018 American Community Survey.^25^ Variables were transformed for interpretability.

### Inclusion and Exclusion Criteria

Patients were included if they had colorectal adenocarcinoma diagnosed between January 1, 2010, and December 31, 2018, underwent a primary surgical resection, and had liver metastasis without extrahepatic metastasis (eTable 2 in Supplement 1). Among this cohort, patients who underwent a nonprimary surgical procedure of distant site of disease were identified. Since the variable that identifies the site of metastasis (Extent of Disease SEER Combined Mets at DX-liver) was only available starting in 2010, year of diagnosis was restricted to 2010 to 2018. Counties that had 10 or fewer patients with CRLM were suppressed to maintain privacy.

For comparison purposes, we included patients with stage I colorectal cancer (CRC) diagnosed between January 1, 2010, and December 31, 2018 (eTable 2 in Supplement 1). Counties that had 10 or fewer patients with stage I CRC were suppressed to maintain privacy. The SEER Research Plus data attributed patients to the county where they resided.

### Statistical Analysis

All analyses were performed at the county level and not at the patient level. The outcome was the county-level proportion of patients diagnosed with CRLM who received a liver metastasectomy. County-level bivariable and multivariable binomial logistic regression models accounting for clustering of outcomes within a county via an overdispersion parameter were used to estimate the county-level odds of receiving a liver metastasectomy for CRLM associated with a 10% increase in poverty rate. The binomial logistic regression model accounting for clustering within a county is equivalent to a logistic regression model with a random effect for county. We chose a logistic regression over linear regression because the odds ratio (OR) is more interpretable than the rates difference. Multivariable models were adjusted for county-level sociodemographic characteristics (eg, population density per square kilometer, residential segregation, percentage of uninsured adults, income inequality, and percentage of the county population aged 65 years or older) and for county-level patient (eg, sex, race and ethnicity, and age group) and tumor (eg, tumor size of primary tumor and nodal status of primary tumor) characteristics. County-level sociodemographic characteristics were adjusted for in the model because they could be associated with the exposure (ie, poverty rate of county of residence) and outcome of interest (ie, undergoing liver surgical resection of CRLM). The same is true for the county-level patient and tumor characteristics: each of these variables can also be associated with the exposure and the outcome of interest. For example, patients residing in counties with higher poverty rates could be of a certain race and ethnicity or age group, have larger tumors, and have nodal involvement of their primary colon cancer. Similarly, these patients could also be less likely to undergo a liver surgical resection for CRLM. Multicollinearity between county-level sociodemographic characteristics was tested using a variance inflation factor and none were colinear. All analyses excluded counties with missing data for rates of surgery and county-level sociodemographic data. Patient race and ethnicity were identified and reported in the SEER Research Plus data as follows: Hispanic (all races), non-Hispanic Black (hereinafter *Black*), non-Hispanic White (hereinafter *White*), or other (non-Hispanic American Indian or Alaska Native, non-Hispanic Asian or Pacific Islander, and non-Hispanic unknown race or ethnicity). For comparison purposes, the analysis was repeated to evaluate the odds of undergoing surgical resection for stage I CRC at the county level according to an increase in county-level poverty rates by 10%.

#### Additional Analysis

An additional analysis was performed using county-level median household income, a potentially more easily understood socioeconomic metric compared with county-level poverty rate, from the Area Health Resources Files.^26^ The year 2014 county-level median household income was used since it fell in the middle of the 2010 to 2018 time period. County-level median household income was scaled by 10 000 to evaluate the association between undergoing a liver metastasectomy for CRLM and for stage I CRC, respectively, at the county level according to an increase in median household income by $10 000. The model was adjusted for all county-level sociodemographic characteristics (except for poverty rate, since this is colinear with median household income).

#### Comparing County-Level Variation in Surgical Rates

Variation in county-level rates of liver metastasectomy for CRLM and surgery for stage I CRC was compared using the *F* statistic and reported graphically using jitter scatter plots overlaid with violin plots. This allowed for visualization of the variability and distribution in rates of liver metastasectomy for CRLM and rates of surgical resection for stage I CRC. County outliers were identified as counties with rates of liver metastasectomy and rates of surgery for stage I CRC, respectively, that were 1.5 times outside of the IQR with respect to the upper and lower quartiles, respectively.

Surveillance Research Program SEER*Stat software, version 8.3.9.2 (National Cancer Institute), was used to extract the data.^27^ Statistical analysis was performed using Stata/IC, version 16.1 (StataCorp LLC). Data analysis was performed on March 2, 2022.

## Results

This cross-sectional study included 11 348 patients who resided in 194 US counties. At the county level, the majority of the population was male (mean [SD], 56.9% [10.2%] vs 43.1% [10.2%] female) and aged between 50 and 64 (38.1% [11.0%]) or 65 and 79 (33.6% [11.4%]) years (Table 1). The mean (SD) population race and ethnicity was reported as Black (15.1% [18.2%]), Hispanic (7.9% [11.3%]), White (71.9% [20.0%]), or other (5.0% [9.4%]). We excluded 3 counties that had a total of 44 patients, since these counties had missing data for county-level sociodemographic variables. The majority of primary colorectal tumors were T3 (mean [SD], 40.3% [10.3%]) or T4 (23.1% [8.9%]), with N1 (26.4% [8.8%]) or N2 (28.9% [9.0%]) nodal disease. The mean county-level rate of liver metastasectomy over all patients with CRLM was 23.7% (10.2%). The only county-level patient and tumor characteristic that was associated with odds of undergoing a liver surgical resection for CRLM was Hispanic ethnicity (OR, 0.39 [95% CI, 0.23-0.66]; *P* = .001) (eTable 3 in Supplement 1).

**Table 1.** Patient Demographic and Tumor Characteristics^a^

Characteristic	Values
Sex	
Female	43.1 (10.2)
Male	56.9 (10.2)
Race and ethnicity	
Hispanic (all races)	7.9 (11.3)
Non-Hispanic	
Black	15.1 (18.2)
White	71.9 (20.0)
Other^b^	5.0 (9.4)
Age, y	
15-49	16.9 (0.8)
50-64	38.1 (11.0)
65-79	33.6 (11.4)
≥80	11.4 (6.8)
Size of primary tumor	
T0/T1/T2	2.2 (2.7)
T3	40.3 (10.3)
T4	23.1 (8.9)
Unknown	33.2 (10.1)
Nodal status of primary tumor	
N0	12.8 (7.1)
N1	26.4 (8.8)
N2	28.9 (9.0)
Unknown	31.9 (10.1)
Liver metastasectomy receipt	23.7 (10.2)

^a^ Data are presented as the mean (SD) percentage of 11 348 patients from 194 US counties. We only included counties in which more than 10 patients had colorectal liver metastasis.

^b^ Includes American Indian or Alaska Native, Asian or Pacific Islander, and unknown race and ethnicity.

According to county-level sociodemographic characteristics, the median county-level percentage below the poverty rate was 15.6% (IQR, 11.2%-19.7%) (Table 2). In 2015, the median percentage of uninsured adults in the included counties was 12.6% (IQR, 8.1%-18.1%).

**Table 2.** Sociodemographic Characteristics of the Study Population^a^

Characteristic (year)	Median (IQR)
Below poverty rate (2010), %	15.6 (11.2-19.7)
Median income (2014), $	51 949 (42 985-62 937)
Population density (2014-2018), per km^2^	106.2 (40.8-268.2)
Residential segregation, Black/White index (2012-2016)	48.3 (39.7-57.6)
Uninsured adults (2015), %	12.6 (8.1-18.1)
Income inequality^b^ (2012-2016)	4.7 (4.2-5.2)
Population aged 65 years or older (2016), %	14.6 (12.9-17.0)

^a^ Data are from 194 US counties. We only included counties in which more than 10 patients had colorectal liver metastasis.

^b^ Reported as the ratio of the household income at the 80th percentile to the household income at the 20th percentile within a county.

Bivariable analysis demonstrated that a 10% increase in county-level poverty rate was associated with decreased odds of undergoing a liver metastasectomy among patients with CRLM (OR, 0.88 [95% CI, 0.79-0.99]; *P* = .03) (Table 3). After adjusting for county-level sociodemographic characteristics and patient and tumor characteristics, the odds of undergoing liver metastasectomy among patients with CRLM remained significantly lower in counties that had a higher poverty rate (per 10% increase; OR, 0.82 [95% CI, 0.69-0.96]; *P* = .02) (Table 3). Conversely, undergoing surgical resection for stage I CRC was not associated with county-level poverty rates (OR, 0.97 [95% CI, 0.90-1.04; *P* = .39) (Table 4).

**Table 3.** Bivariable and Multivariable Binomial Logistic Regression Accounting for Overdispersion to Evaluate Odds of Undergoing Liver Surgical Resection for Colorectal Liver Metastasis at the County Level^a^

Characteristic	Univariable		Multivariable^b^	
	OR (95% CI)	*P* value	OR (95% CI)	*P* value
Below poverty rate (increase by 10%), %	0.88 (0.79-0.99)	.03	0.82 (0.69-0.96)	.02
Population density (increase by 1000), per km^2^	0.97 (0.92-1.03)	.003	0.99 (0.91-1.07)	.79
Residential segregation, Black/White index (increase by 10%)	1.02 (0.97-1.07)	.42	1.00 (0.94-1.07)	.90
Uninsured adults (increase by 10%), %	1.02 (0.90-1.15)	.77	1.05 (0.88-1.25)	.60
Income inequality	0.95 (0.88-1.03)	.24	1.00 (0.88-1.14)	.99
Population aged 65 years or older (increase by 10%), %	0.97 (0.78-1.19)	.75	0.86 (0.65-1.13)	.29

Abbreviation: OR, odds ratio.

^a^ Data are from 194 US counties. We only included counties in which more than 10 patients had colorectal liver metastasis. Binomial logistic regression, count of liver surgical resection/count of colorectal liver metastasis at the county level.

^b^ Adjusted for county-level covariates of interest and county-level patient and tumor characteristics.

**Table 4.** Multivariable Binomial Logistic Regression Accounting for Overdispersion to Evaluate Odds of Undergoing Surgical Resection at the County Level^a^

Characteristic	Odds ratio (95% CI)	*P* value
Colorectal liver metastasis (194 counties)		
Below poverty rate (increase by 10%), %	0.79 (0.67-0.92)	.003
Median household income (per $10 000), $	1.06 (1.01-1.11)	.02
Stage I colorectal cancer		
Below poverty rate (increase by 10%), % (371 counties)	0.97 (0.90-1.04)	.39
Median household income (per $10 000), $ (330 counties)	1.01 (0.98-1.03)	.56

^a^ Binomial logistic regression, count of surgical resection/count of cancer at the county level, and adjusted for all covariates of interest (population density, residential segregation Black/White index, percentage of uninsured adults, income inequality, and percentage of county population aged 65 years or older).

In the model evaluating the association between odds of undergoing liver metastasectomy and county-level poverty rates, we adjusted for the county-level rate of surgical resection for stage I CRC to control for a potentially additional measure of care seeking, and the findings did not change substantially.

The odds of undergoing liver metastasectomy was higher in counties with a higher 2014 median household income (per $10 000 increase; OR, 1.06 [95% CI, 1.01-1.11]; *P* = .02) (Table 4). Conversely, undergoing surgical resection for stage I CRC was not associated with median household income (per $10 000 increase; OR, 1.01 [95% CI, 0.98-1.03]; *P* = .56).

Despite the difference in rates of surgery (mean county-level rates were 0.24 for liver metastasectomy for CRLM and 0.75 for surgery for stage I CRC), the variance at the county level for these 2 surgical procedures was similar (*F*_370, 193_ = 0.81; *P* = .08) (Figure). County outliers for surgery for stage I CRC were predominantly at the lower end of the distribution, while those for liver metastasectomy for CRLM were at the upper end of the distribution.

**Figure.**
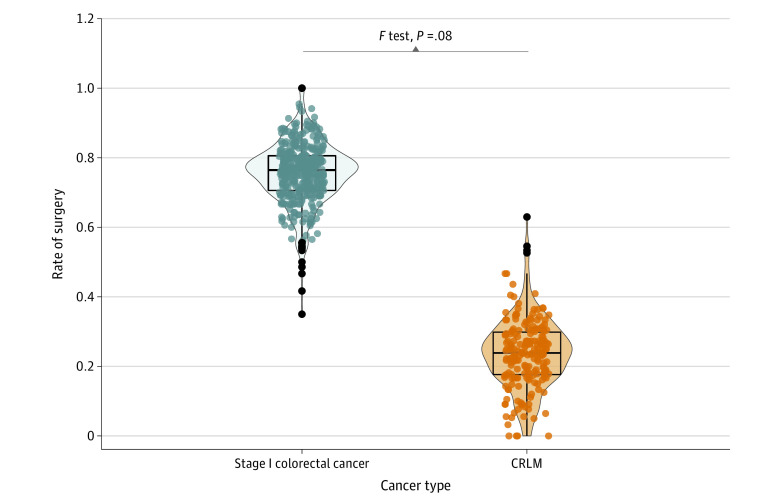
Variation and Distribution of County-Level Rates of Liver Metastasectomy for Colorectal Liver Metastasis and County-Level Rates of Surgical Resection for Stage I Colorectal Cancer The *F* statistic was used to compare the variance in county-level surgical rates for colorectal liver metastasis (CRLM) and stage I colorectal cancer.

## Discussion

The findings of this cross-sectional study suggest that patients residing in US counties with a greater proportion of residents with incomes below the federal poverty line were less likely to undergo liver metastasectomy for CRLM; however, the same was not true for undergoing surgery for stage I CRC. These findings might be explained by the complex nature of care and need for multidisciplinary specialists that are required to treat patients with CRLM. Access to surgical care for complex gastrointestinal cancers is likely facilitated or hindered by multiple intersecting and difficult-to-measure social determinants of health and population-level measures of wealth,^28,29^ factors that might be reflected in the proportion of residents living in a county whose income is below the federal poverty level. Other factors that might affect access to liver metastasectomy for CRLM include patient-level factors, such as financial toxicity and lack of trust in the health care system and practitioners, and system-level factors like accessibility of specialized hospitals and expertise in regions of poverty. These findings add to the literature and provide a deeper understanding of a patient-level factor (where a patient resides) that may affect whether patients with potentially resectable CRLM are treated with liver surgical resection. Potential solutions to address unequal access to liver metastasectomy for CRLM should consider not only the hospital where patients seek care but also where they reside, since the latter may affect where care is ultimately provided and what kind of care patients receive.

Previous studies have focused on access to liver surgical resection for CRLM at the patient and hospital levels. There is substantial variation in these studies in terms of whether patients with CRLM are considered or offered curative-intent liver surgical resection in different populations and at different centers.^2,10,14,30^ Additionally, there is variation in how patients are treated among hepatobiliary surgeons.^31^ However, none of these previous studies examined regional variation. Reporting on regional variation is important since it may inform future interventions that are population focused rather hospital focused, and it also highlights the importance of adapting treatment and screening based on individual and community-level factors, which has been previously proposed.^32^ Although interventions to address social determinants of health are more complex to design and implement, addressing these issues may be essential in improving access to care for various complex gastrointestinal cancers.

Regional variations in how CRLM outside the US is treated and how other cancers are treated has increasingly been reported in the literature. In the Netherlands, interregional practice variations in the use of local therapy for synchronous CRLM have been found, although these interregional practice variations have recently been decreasing.^30^ Despite the centralization of care, Fenton et al^33^ reported substantial variation in rates of liver metastasectomy across hospitals in the UK National Health Service (NHS) between 2009 and 2012. Additionally, Vallance et al^34^ reported that patients diagnosed at hepatobiliary surgical units were notably more likely to undergo a liver metastasectomy compared with patients diagnosed at hospital trusts in the NHS. In the treatment of breast cancer in the US, regional variation in the use of surgery for stage I breast cancer, chemotherapy, radiation treatment, and endocrine therapy has been reported.^35^ Although we found that liver metastasectomy was performed less frequently for CRLM than surgery was performed for stage I CRC, the variation across US counties in rates of surgery for these disease entities was not different. This finding may underscore that both surgical interventions are facilitated or hindered in their use by similar regional factors, including geographic distance and accessibility. This is important since recently there has been a trend of a greater proportion of patients living farther away from American College of Surgeons cancer programs (for 2015 vs 2005).^36^

Variation in surgical care is not a novel concept.^37,38,39^ However, to our knowledge, it has not been reported previously for liver metastasectomy for CRLM—a condition that is curable but requires a highly coordinated oncology team with access to subspecialist surgical care. The results of this study suggest that patients from counties with greater poverty undergo potentially curative resection for CRLM less frequently. The next steps in our work will focus on negative and positive outlier counties to assess if there are interventions that could be designed and implemented in counties where liver metastasectomy for CRLM is being underutilized. This could inform the development of guidance regarding the appropriate geographic distribution of surgeons trained in evaluating and treating patients with CRLM, which could model work done in studying equity vs efficiency in the geographic placement of health care services in low- and low- to middle-income countries.^40^ Additionally, another possible solution could be to strengthen collaborations and improve the ease with which patients from one county or health care system access care in a different county or health care system. Patient navigation programs, such as the National Cancer Institute Patient Navigator Research Program, have been associated with eliminating disparities in delays in “diagnostic resolution,” which the authors defined as the time between initial screening abnormality and final definitive diagnostic or evaluation, among patients with lower income.^41^ County- and state-level interventions may improve not only access to liver metastasectomy for CRLM but also access to surgical care for other complex gastrointestinal cancers.

### Limitations

There are certain limitations to this cross-sectional study that should be considered when interpreting the results. First, the SEER Research Plus database is retrospective, and findings using these data cannot be reported as being causal. Our cross-sectional design in which rates of surgical resection, CRLM, and sociodemographic characteristics were assessed concurrently limits our ability to make causal claims. However, it is more likely that county-level sociodemographic characteristics precede the cancer diagnosis, rather than the reverse. Second, our ecological design relies on county-level SEER data and we cannot identify patient-specific data, so patterns reported here may not hold when individual-level data are considered. However, this is a first step toward identifying places with poorer access to care. Third, how patients access cancer care is not always defined by the county in which they live. Patients may cross county lines when accessing care. An alternative study design would be to use health service area data, but there are no sociodemographic data at the health service area that we could have used to perform this analysis. Fourth, the SEER Research Plus database does not provide data on the number of liver metastases or whether there was bilobar disease, which would provide information on whether a liver metastasectomy was possible. However, there should be no substantial differences in rates of surgically resectable CRLM at the county level. Fifth, we needed to exclude counties with fewer than 10 patients to maintain privacy per requirements of the SEER data use agreement. The impact of not including counties with less than 10 patients in the analysis is most likely not substantial, since the numbers are so small. As such, our analysis is more generalizable to counties that have 10 patients or more. Sixth, we did not include a trend analysis in the model since it is not feasible because the county-level exposure of interest (ie, county-level poverty) is not available at the yearly level. Additionally, the 9-year timeframe is relatively short and there is most likely not substantial variation from year to year.

## Conclusions

County-level liver metastasectomy for CRLM was associated with poverty level in the US. However, surgery for a more common and less complex cancer comparator (ie, stage I CRC) was not associated with county-level poverty rates. These findings may highlight that access to surgical care for complex gastrointestinal cancers, like CRLM, may be partially affected by where patients live. County-level poverty rates may serve as a proxy for difficult-to-measure social determinants of health that impact the care that patients with CRLM receive. Future interventions that are designed to address unequal access to liver metastasectomy for CRLM should consider where patients reside and potential social determinants of health.

## Notes

Supplement 1. **eTable 1.** Description of County Sociodemographic Variables **eTable 2.** Histology and Surgical Procedure Codes for Colon, Rectosigmoid, and Rectal Adenocarcinoma **eTable 3.** Univariable Binomial Logistic Regression Accounting for Overdispersion to Evaluate Odds of Undergoing Liver Surgical Resection for Colorectal Liver Metastasis at the County Level for 194 Counties

Supplement 2.
Data Sharing Statement
