# Supplementary material for: Association of Variation in US County-Level Rates of Liver Surgical Resection for Colorectal Liver Metastasis With Poverty Rates in 2010
Source: JAMA Netw Open. 2023 Feb 27;6(2):e230797. doi: 10.1001/jamanetworkopen.2023.0797 (PMC9972196; doi:10.1001/jamanetworkopen.2023.0797)
Supplement: Supplement 1. — eTable 1. Description of County Sociodemographic Variables eTable 2. Histology and Surgical Procedure Codes for Colon, Rectosigmoid, and Rectal Adenocarcinoma eTable 3. Univariable Binomial Logistic Regression Accounting for Overdispersion to Evaluate Odds of Undergoing Liver Surgical Resection for Colorectal Liver Metastasis at the County Level for 194 Counties [file jamanetwopen-e230797-s001.pdf]

Supplementary Online Content

Molina G, Ruan M, Lipsitz SR, et al. Association of variation in US county-level rates of liver surgical resection for colorectal liver metastasis with poverty rates in 2010. *JAMA Netw Open*. 2023;6(2):e230797. doi:10.1001/jamanetworkopen.2023.0797

eTable 1. Description of County Sociodemographic Variables

eTable 2. Histology and Surgical Procedure Codes for Colon, Rectosigmoid, and Rectal Adenocarcinoma

eTable 3. Univariable Binomial Logistic Regression Accounting for Overdispersion to Evaluate Odds of Undergoing Liver Surgical Resection for Colorectal Liver Metastasis at the County Level for 194 Counties

This supplementary material has been provided by the authors to give readers additional information about their work.

© 2023 Molina G et al. *JAMA Network Open*.

eTable 1. Description of County Sociodemographic Variables

**Measure**	
2010 Percent Below Poverty Rate, median (IQR)	• Number of people in living in poverty out of total people within a county, following the US Census Bureau's definition of poverty<br>• Poverty is defined using the US Census Bureau's poverty guidelines, which uses a set of pre-tax income thresholds that vary by family size and composition; these thresholds are adjusted for inflation (using the Consumer Price Index) but do not vary based on geographical location<br><br>https://www.countyhealthrankings.org/explore-health-rankings/measures-data-sources/county-health-rankings-model/health-factors/social-and-economic-factors/income/<br>https://www.census.gov/topics/income-poverty/poverty/guidance/poverty-measures.html
2014-2018 Population Density by County	• Average number of residents per square kilometer within a county; data obtained from the US Census Bureau's American Community Survey<br><br>https://covid19.census.gov/datasets/21843f238cbb46b08615fc53e19e0daf_1/explore?location=37.334265%2C-108.856411%2C5.00
2012-2016 Residential Segregation – Black/White Index, median (IQR)	• An index generated from census data to compare how evenly distributed white and black residents are dispersed within a county. The index ranges on a scale from 0 (complete integration) to 100 (complete segregation)<br>• Counties are given "missing" designation on the database if there are less than 100 black residents living there in the specified time period; 65% of US counties have more than 100 black residents<br><br>https://www.countyhealthrankings.org/explore-health-rankings/measures-data-sources/county-health-rankings-model/health-factors/social-and-economic-factors/family-social-support/residential-segregation-blackwhite
2015 Percent of Uninsured Adults, median (IQR)	• For adults ages 18-64 (before the Medicare age), the percentage of adults within a county with no health insurance coverage (private, employee, or Medicaid coverage)<br>• Data obtained through the US Census Bureau's Small Area Health Insurance Estimates (SAHIE) program<br><br>https://www.countyhealthrankings.org/explore-health-rankings/measures-data-sources/county-health-rankings-model/health-factors/clinical-care/access-to-care/uninsured-adults
2012-2016 Income Inequality	• Income inequality is reported as the ratio of the household income at the 80th percentile to the household income at the 20th percentile within a county<br>• Income is defined as being the sum of all reported amounts of wage or salary income for a household, excluding income from capital gains or lump-sum receipts<br><br>https://www.countyhealthrankings.org/explore-health-rankings/measures-data-sources/county-health-rankings-model/health-factors/social-and-economic-factors/income/income-inequality
2016 Percent of County Population that is 65 and older, median (IQR)	• Number of residents 65 years old or older out of total residents within a county; data obtained from Census population estimates<br><br>https://www.countyhealthrankings.org/2022-measures

© 2023 Molina G et al. *JAMA Network Open*.

eTable 2. Histology and Surgical Procedure Codes for Colon, Rectosigmoid, and Rectal Adenocarcinoma

**Histology Codes**	**Description**
8140	Adenocarcinoma, NOS
8144	Adenocarcinoma, intestinal type
8210	Adenocarcinoma, in adenomatous polyp
8220	Adenocarcinoma, in adenomatous polyposis coli
8255	Adenocarcinoma, with other types of carcinoma
8261	Adenocarcinoma, in villous adenoma
8263	Adenocarcinoma, in tubulovillous adenoma
8574	Adenocarcinoma, with neuroendocrine differentiation
**Procedure Codes**	**Description**
Colon	
30	Partial colectomy, segmental resection
32	Plus resection of contiguous organ; example: small bowel, bladder
40	Subtotal colectomy/hemicolectomy (total right or left colon and a portion of transverse colon)
41	Plus resection of contiguous organ; example: small bowel, bladder
50	Total colectomy (removal of colon from cecum to the rectosigmoid junction; may include a portion of the rectum)
51	Plus resection of contiguous organ; example: small bowel, bladder
60	Total proctocolectomy (removal of colon from cecum to the rectosigmoid junction, including the entire rectum)
61	Plus resection of contiguous organ; example: small bowel, bladder
70	Colectomy or coloproctotectomy with resection of contiguous organ(s), NOS
80	Colectomy, NOS
Rectum and Rectosigmoid Junction	
30	Wedge or segmental resection; partial proctosigmoidectomy, NOS
31	Plus resection of contiguous organs; example: small bowel, bladder
40	Pull through WITH sphincter preservation (colo-anal anastomosis)
50	Total proctectomy
51	Total colectomy
55	Total colectomy WITH ileostomy, NOS
56	Ileorectal reconstruction
57	Total colectomy WITH other pouch; example: Koch pouch
60	Total proctocolectomy, NOS
65	Total proctocolectomy WITH ileostomy, NOS
66	Total proctocolectomy WITH ileostomy and pouch
70	Colectomy or proctocolectomy resection in continuity with other organs; pelvic exenteration
80	Colectomy, NOS; Proctectomy, NOS

© 2023 Molina G et al. *JAMA Network Open*.

eTable 3. Univariable Binomial* Logistic Regression Accounting for Overdispersion to Evaluate Odds of Undergoing Liver Surgical Resection for Colorectal Liver Metastasis at the County Level for 194 Counties

Patient Characteristics		**OR**	**95% CI**	**P-value**
Sex				
	Female	**Reference**		
	Male	0.93	0.40-2.19	0.87
Age Group				
	15-49	1.03	0.33-3.23	0.96
	50-64	0.80	0.32-1.98	0.62
	65-79	**Reference**		
	≥ 80	0.51	0.15-1.74	0.28
Race/Ethnicity				
	Hispanic (All Races)	0.39	0.23-0.66	0.001
	Non-Hispanic Black	1.03	0.72-1.46	0.88
	Non-Hispanic Other**	0.70	0.43-1.12	0.13
	Non-Hispanic White	**Reference**		
Tumor Size of Primary Tumor				
	T0/T1/T2	8.9	0.81-97.6	0.07
	T3	1.69	0.61-4.72	0.31
	T4	**Reference**		
	Unknown	1.36	0.47-3.95	0.57
Nodal Status of Primary Tumor				
	N0	0.45	0.12-1.74	0.25
	N1	2.09	0.65-6.77	0.22
	N2	**Reference**		
	Unknown	0.95	0.33-2.74	0.92

*Binomial logistic regression, count of liver surgical resection / count of colorectal liver metastasis at the county level
**Non-Hispanic Other includes Non-Hispanic American Indian/Alaska Native, Non-Hispanic Asian or Pacific Islander, and Non-Hispanic unknown race.

© 2023 Molina G et al. *JAMA Network Open*.
