# Supplementary material for: Association of Variation in US County-Level Rates of Liver Surgical Resection for Colorectal Liver Metastasis With Poverty Rates in 2010
Source: JAMA Netw Open. 2023 Feb 27;6(2):e230797. doi: 10.1001/jamanetworkopen.2023.0797 (PMC9972196; doi:10.1001/jamanetworkopen.2023.0797)
Supplement: Supplement 2. — Data Sharing Statement [file jamanetwopen-e230797-s002.pdf]

Data Sharing Statement

Molina. Association of Variation in US County-Level Rates of Liver Surgical Resection for Colorectal Liver Metastasis With Poverty Rates in 2010. *JAMA Netw Open*. Published February 27, 2023. doi:10.1001/jamanetworkopen.2023.0797

Data
Data available: No

Additional Information
Explanation for why data not available: The SEER Research Plus Dataset is a publicly available dataset.
